# A neurorestorative approach to Parkinson's disease

**DOI:** 10.18632/oncotarget.17108

**Published:** 2017-04-14

**Authors:** Giovanni Diana, Maria Teresa Ciotti, Marco Musilli

**Affiliations:** Department of Therapeutic Research and Medicines Evaluation, Istituto Superiore di Sanità, Regina Elena 299, Roma, Italy

**Keywords:** Parkinson's disease, neurorestoration, Rho GTPases, human microbiota, E. coli

Parkinson's disease (PD) is the second most common neurodegenerative disorder. Its pathologic hallmark consists in the degeneration of substantia nigra dopaminergic (DA-ergic) neurons projecting to the striatum and resulting deficit in striatal DA neurotransmission. In spite of extensive research, the etiopathogenesis of PD is not yet clearly understood. This poor understanding is reflected in the lack of treatments that can slow or reverse the disease. Current therapy aims at restoring DA neurotransmission. This goal is achieved with DA precursors, such as l-DOPA, drugs reducing catabolism of endogenous DA, thereby increasing the availability of DA in the synaptic cleft and DA receptor agonists. All these treatments are merely symptomatic and do not modify the evolution of PD.

Experimental treatments intended to stop and possibly reverse the neural degeneration of the nigrostriatal pathway have been proposed. Neurotrophic factors promote the survival, differentiation and maintenance of neurons in adult vertebrate nervous system. Therefore, treating degenerating neurons with these molecules might promote their survival and activity. Neurotrophic factors are effective in restoring neurochemical deficits and correcting behavioral changes in PD models. However, they exhibit delivery issues, low efficacy and safety concerns in clinical settings [1].

In a recent work, Musilli et al. targeted cerebral Rho GTPases in a rat model of PD obtained by unilateral injection of 6-hydroxydopamine (6-OHDA) in the striatum [2]. Rho GTPases are a class of regulatory proteins encompassing Rho, Rac and Cdc42 subfamilies that govern cell morphology and act as effectors of neurotrophic factors [3]. Modulation of Rho GTPase signaling might thus mimic the effects of neurotrophic factors and disclose promising therapeutic opportunities. However, little evidence exists of such a beneficial action. Rho GTPases are involved in a number of cell functions, including vesicle exocytosis, apoptosis, gene transcription. Not surprisingly, the derangement of their signaling has been linked to several disorders. At the level of CNS, it has been consistently associated with intellectual disability [4]. Some reports also suggested a potential involvement in parkinsonism [5].

Authors showed that Cytotoxic Necrotizing Factor 1 (CNF1), a bacterial protein toxin produced by some strains of *E. coli* that permanently activates Rho, Rac and Cdc42 in eukaryotic cells, exerts neurotrophic effects in cultured DA neurons. The administration of CNF1 *in vivo* activates striatal Rac and Cdc42 and corrects behavioural asymmetries and the neurochemical and trophic imbalance caused by unilateral brain injection of 6-OHDA. In the aforementioned experiments, the damage of the nigrostriatal system was long-standing (14 or 25 weeks) at the time of CNF1 treatment. Therefore, the modulation of Rho signaling might promote the recovery of the damaged nigrostriatal pathway and not only slow its degeneration. For that reason, the therapeutic approach described by Musilli et al. might be effective in the advanced stages of the disease, when the loss of DA cells is severe and PD is poorly controlled by the available therapies [6].

Authors concluded that CNF1 is endowed with neurotrophic effects on substantia nigra DA-ergic neurons and shows preclinical efficacy in a model of PD. The effects depend on the activation of Rho GTPases, as the recombinant molecule CNF1 C866S, in which the enzymatic activity was suppressed by replacing serine to cysteine at position 866, is ineffective. Targeting Rac and Cdc42 signaling might thus represent a promising restorative-regenerative approach to the treatment of PD and possibly of other conditions characterized by neuronal loss or degeneration.

Environmental factors are believed to play a substantial role in the development of PD. Among them, microbial agents may contribute to the etiopathogenesis of the disease. A recent study suggested that gut microbiota is linked to PD pathology [7]. However, hypotheses as to how microbes might exert this action are still inconclusive. Like CNF1, a number of bacterial proteins influence Rho GTPase signaling, either activating or depressing it [8]. The study by Musilli et al. provides a putative mechanism by which microbial products may affect the occurrence and course of PD.
